# The effects of breath-holding on pulmonary regurgitation measured by cardiovascular magnetic resonance velocity mapping

**DOI:** 10.1186/1532-429X-11-1

**Published:** 2009-01-14

**Authors:** Bengt Johansson, Sonya V Babu-Narayan, Philip J Kilner

**Affiliations:** 1 Cardiovascular Magnetic Resonance Unit, Royal Brompton Hospital, Sydney Street, SW3 6NP, London, UK

## Abstract

**Background:**

Pulmonary regurgitation is a common and clinically important residual lesion after repair of tetralogy of Fallot. Cardiovascular magnetic resonance (CMR) phase contrast velocity mapping is widely used for measurement of pulmonary regurgitant fraction. Breath-hold acquisitions, usually acquired during held expiration, are more convenient than the non-breath-hold approach, but we hypothesized that breath-holding might affect the amount of pulmonary regurgitation.

**Methods:**

Forty-three adult patients with a previous repair of tetralogy of Fallot and residual pulmonary regurgitation were investigated with CMR. In each, pulmonary regurgitant fraction was measured from velocity maps transecting the pulmonary trunk, acquired during held expiration, held inspiration, by non-breath-hold acquisition, and also from the difference of right and left ventricular stroke volume measurements.

**Results:**

Pulmonary regurgitant fraction was lower when measured by velocity mapping in held expiration compared with held inspiration, non-breath-hold or stroke volume difference (30.8 *vs*. 37.0, 35.6, 35.4%, *p *= 0.00017, 0.0035, 0.026). The regurgitant volume was lower in held expiration than in held inspiration (41.9 *vs*. 48.3, *p *= 0.0018). Pulmonary forward flow volume was larger during held expiration than during non-breath-hold (132 *vs*. 124 ml, *p *= 0.0024).

**Conclusion:**

Pulmonary regurgitant fraction was significantly lower in held expiration compared with held inspiration, free breathing and stroke volume difference. Altered airway pressure could be a contributory factor. This information is relevant if breath-hold acquisition is to be substituted for non-breath-hold in the investigation of patients with a view to re-intervention.

## Background

Over the last decades the prognosis in tetralogy of Fallot has improved dramatically. The long term survival after surgical repair is excellent [1,2]. There is, however, late mortality and morbidity related to regurgitation of the reconstructed right ventricular outflow tract. In this setting, pulmonary regurgitation has been found to predispose to arrhythmia and right ventricular dysfunction [3-6]. Its association with increased risk of sudden death makes the assessment of pulmonary regurgitation important in the follow-up and management of this patient group. Significant pulmonary regurgitation may also be found in patients who have undergone pulmonary valvotomy for isolated pulmonary stenosis, and those with congenital absence of the pulmonary valve.

Cardiovascular magnetic resonance (CMR) provides a valuable, arguably the best available tool for quantification of pulmonary regurgitation. Non-breath-hold velocity acquisitions during free breathing have been used as a gold standard for evaluation of pulmonary regurgitation [7,8]. Faster velocity mapping sequences that can be acquired during a single breath-hold have been introduced in recent years. It has been shown that the pulmonary regurgitant volume measured by a real time conductance catheter technique in intubated patients increases with increased airways pressure, so much so that "the subtle waxing and waning in mean airway pressure associated with positive pressure ventilation also led to significant changes in pulmonary incompetence" [4]. We hypothesised that the breath-holding used in CMR acquisitions might affect the amount of pulmonary regurgitation. In this study we compared measurements of pulmonary regurgitant fraction by velocity maps acquired during free breathing, held inspiration and held expiration, and those calculated from right and left ventricular stroke volume difference.

## Materials and methods

### Patients

Forty-three adult patients (18 females) with repaired tetralogy of Fallot were prospectively recruited from a dedicated Adult Congenital Heart Unit. Inclusion criteria were previous surgical repair of tetralogy of Fallot and clinical stability, without any significant residual shunt, pulmonary regurgitation with a regurgitant fraction > 5% and no contraindication for CMR. Patients with more than trivial regurgitation of the atrioventricular or aortic valves were excluded from the calculation of pulmonary regurgitant fraction by stroke volume difference. The mean age of the patients was 30.6 ± 9.5 and the anatomical reconstruction was performed at a mean age of 5.5 ± 5.6 years. All patients gave their written informed consent. The study was approved by the research ethics committee, Royal Brompton Hospital.

### CMR

All patients were investigated in a 1.5 T Siemens Sonata MR scanner (Siemens, Erlangen, Germany). The scans included assessment of anatomy using contiguous stacks of 7 mm thickness multislice scout images in transaxial, coronal and sagittal orientations prior to acquisition of balanced steady state free precession (bSSFP) cine images and phase contrast velocity maps, as described below.

### Ventricular volumes

A stack of bSSFP cine images was acquired in short axis planes at 10 mm intervals from the atrio-ventricular junction to the apex, located with respect to two- and four-chamber long axis cines. These cines were acquired during held expiration. End diastolic and end systolic volumes of both ventricles were measured by planimetry using Simpson's method using appropriate software (CMRtools, Cardiovascular Imaging Solutions, London, UK). A single observer (SBN) performed all measurements as previously described [9].

### Pulmonary regurgitation, and stenosis if present

**Figure 1:**
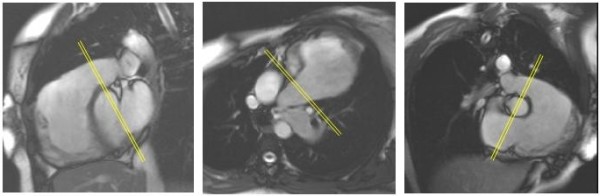
**The cine images of the right ventricular outflow tract and the pulmonary trunk show the alignment of the phase velocity mapping plane (indicated by double lines)**. The sagittal view (left) was acquired at end inspiration as well as end expiration to be used for alignment of the respective velocity acquisition. The middle image is an oblique transaxial plane and the right image an oblique inlet and outlet view of the right ventricle.

Transaxial scout images were used to locate an oblique sagittal bSSFP cine aligned with the right ventricular outflow tract as shown in Figure 1 (left), with further oblique cines showing the pulmonary trunk (Figure 1, centre and right) aligned relative to this and other preceding images. Pulmonary regurgitation was quantified by non-breath-hold, inspiratory breath-hold and expiratory breath-hold acquisitions, using retrospectively gated Maxwell corrected phase contrast velocity mapping sequences with the following parameters. Breath-hold acquisitions used 5–7 segments, echo time 2.2 ms, flip angle 27°, 695 Hz/pixel, 8 mm slice thickness, 256 image matrix at 65% phase resolution giving 1.3 × 2.0 mm pixel size. Non-breath-hold acquisitions were not segmented, flip angle 30°, 235 Hz/pixel, 8 mm slice thickness, 256 image matrix at 80% phase resolution giving 1.3 × 1.7 mm pixel size. The acquisition window for each reference and velocity encoded data pair was 49 ms for breath-hold and 28 ms for non-breath-hold. Both types of acquisition were reconstructed as 20 frames per cardiac cycle according to the manufacturer's routine post-processing algorithm.

Velocity was encoded through a plane transecting the pulmonary trunk immediately downstream of the expected pulmonary valve level and proximal to the bifurcation (Figure 1). This plane was adjusted to the same anatomical level relative to scout cine acquisitions of the right ventricular outflow tract acquired in either held inspiration of held expiration, as appropriate. The held expiratory scout was used to locate the non-breath-hold velocity acquisition. A velocity encoding range just sufficient to avoid aliasing was chosen, typically 2 m/s, but higher, up to 3.5 m/s, if there was a degree of valvular or supravalvular stenosis. The peak systolic velocity through this suprapulmonary plane was measured in the expiratory breath-hold acquisition, and the pulmonary regurgitant fraction was calculated as the diastolic regurgitant flow volume expressed as a percentage of the forward flow volume for each acquisition [8]. The regurgitant fraction was also calculated, in the absence of other significant heart valve regurgitation, using the stroke volume difference (right ventricular stroke volume - left ventricular stroke volume × 100 ÷ right ventricular stroke volume).

Because of the potential effect of branch stenosis on regurgitation, the multislice scouts were assessed for potential branch pulmonary artery stenoses, and where stenosis could not be excluded, bSSFP cines were aligned with the lumen of any suspected narrow region of the right and left pulmonary artery for qualitative assessment. Attribution of mild, moderate (or severe) degrees of branch stenosis relied on visual assessment of three factors: the degree of luminal narrowing, the formation and narrowness of a resulting jet, and the degree of damping of systolic arterial expansion distally.

### Statistics

Data are presented as means and standard deviations. To compare means a paired *t*-test was used. As multiple comparisons were performed, the *p*-value was corrected according to Bonferroni by multiplying the *p*-value with the number of comparisons. Only *p*-values less than 0.05 were regarded as significant. All calculations were performed using the SPSS 11.5 statistical software (SPSS inc., Chicago, IL, USA).

## Results

### Pulmonary regurgitant fraction

**Figure 2:**
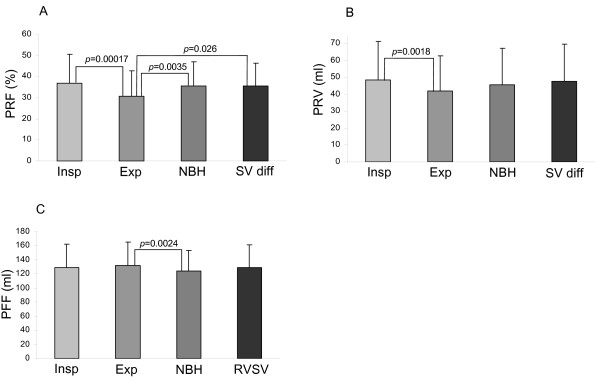
**Bar graphs showing pulmonary regurgitant fraction (PRF, graph A), pulmonary regurgitant volume (PRV, graph B) and pulmonary forward flow volume (PFF, graph C) during held inspiration (Insp), held expiration (Exp), non-breath-hold (NBH) and calculated by planimetric stroke volume difference (SV diff) or planimetric right ventricular stroke volume (RVSV)**. In graph A, the regurgitant fraction during held expiration differs significantly from regurgitant fractions during held inspiration, free breathing and as calculated by the planimetric stroke volume difference.

**Figure 3:**
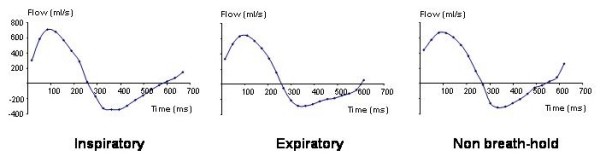
**The three pulmonary flow curves in a typical case are shown above**. In this individual, the pulmonary regurgitant fraction was 57% in held inspiration (left), 38% in held expiration (middle) and 53% during non-breath-hold acquisition (right). Late-diastolic forward flow, coinciding with atrial systole, is seen as positive flow at the right hand end of each curve in this case.

The pulmonary regurgitant fractions calculated by the four different methods are shown in Figure 2A. Pulmonary regurgitant fraction was lower in held expiration compared with held inspiration, free breathing and stroke volume difference (30.8 ± 11.9 *vs*. 37.0 ± 13.6, 35.6 ± 11.4, 35.4 ± 11.0%, *p *< 0.00017, 0.0035, 0.026). There was no significant difference between the pulmonary regurgitant fraction in held inspiration, free breathing and stroke volume difference. A typical example of the flow patterns is shown in Figure 3.

### Pulmonary regurgitant volume

The regurgitant volumes obtained by velocity maps and ventricular stroke volume difference are shown in Figure 2B. The regurgitant volume was lower in held expiration than in held inspiration (41.9 ± 20.9 *vs*. 48.3 ± 22.8, *p *= 0.0018). There was no significant difference between regurgitant volume in held inspiration, free breathing or calculated from stroke volume difference.

### Pulmonary forward flow volume

The pulmonary forward flow volumes obtained by velocity mapping and planimetric right ventricular (RV) stroke volumes are shown in Figure 2C. They were larger during expiratory breath-hold than during non-breath-hold (132.2 ± 33 *vs*. 123.6 ± 30 ml, *p *= 0.0024). There was no significant difference between the forward flow volumes measured in held inspiration or during free breathing, or between either of these and the RV stroke volumes measured by planimetry.

### Pulmonary and pulmonary artery branch stenosis

Evidence of no more than mild residual pulmonary stenosis was identified by analysis of velocities through the suprapulmonary plane indicated in Figure 1. The highest peak velocity recorded was 2.7 m/s. The mean and SD of peak recorded velocities was 1.78 ± 0.34 m/s. In the branch pulmonary arteries, mild left sided stenosis was found in 7, moderate unilateral left sided stenosis in 1, bilateral mild stenosis in 3 and bilateral moderate stenosis in 1.

## Discussion

Pulmonary regurgitation is relatively common after surgery for congenital heart disease with RV outflow obstruction, as is a degree of residual pulmonary stenosis. These are patients in whom serial measurements can inform decisions on management and the consistency and comparability of measurements is important. More rapid and convenient breath-hold acquisitions are likely to be used increasingly for the investigation and follow-up of pulmonary regurgitation. As far as we know, it is common practice for held expiration to be used for breath-hold acquisitions as this has been considered to give a more reproducible and sustained diaphragm position than held inspiration in various imaging situations [10-12]. Children and patients with learning difficulties, however, may find it easier to hold their breath after inspiration, which could potentially lead to a change of acquisition technique between initial and a follow-up studies.

We found that a lower regurgitant fraction was measured when the breath was held at end expiration, and that increased forward flow volume and decreased regurgitant volume both appeared to contribute. The underlying mechanisms remain uncertain, but the marked effect of pulmonary airway pressure on regurgitation reported by Redington [4] is likely to be a key factor. It must be remembered that the pulmonary microvessels are of low resistance and high compliance [13]. Expiratory or inspiratory breath-holds are not, of course, the same as the *processes *of expiration or inspiration. According to our usual clinical practice, we had instructed patients, speaking slowly, either to 'breathe in and hold' or to 'breathe in and breathe out and hold' before the respective acquisitions. Ley et al. investigated the effects of 'large volume inspiratory breath-hold and expiratory breath-hold' velocity acquisitions on pulmonary and aortic flow parameters in healthy volunteers, comparing the results to those acquired during free breathing. They found pulmonary blood flow to be reduced during (deep) inspiratory breath-hold, and less so during expiratory breath-hold, compared with free breathing [14]. We are not aware that the effects of breath-holding on alveolar air pressure have been investigated, however. Held inspiration, if the mouth and glottis are closed and the diaphragm is relaxed, might be expected to result in an increase of alveolar air pressure and so increased pulmonary microvascular resistance, while held expiration might have the opposite effect. If this is the case, the differences of intra-alveolar pressure might explain the differences of pulmonary regurgitant fraction measured by the different acquisition techniques.

The differences between the regurgitant fractions measured by expiratory breath-hold velocity mapping and those by stroke volume difference, which also used expiratory breath-hold acquisitions, must have a different explanation, however. The two approaches do not necessarily measure the same thing. While the former is calculated from the pulmonary regurgitant volume expressed as a percentage of pulmonary forward flow, the latter is calculated from the stroke volume difference expressed as a percentage of the RV stroke volume. The stroke volume difference could be more than the pulmonary regurgitant volume if there were also undetected tricuspid regurgitation, and the RV stroke volume will be less than the pulmonary forward flow if there is late diastolic forward flow, which has been referred to as 'restrictive RV physiology' [15]. As we understand it, this is caused by forward flow from the right atrium to the pulmonary artery through a full and conduit-like right ventricle, with no significant change of RV volume in this phase. These factors may contribute to the higher regurgitant fraction derived by the volumetric compared with the flow method, although methodological errors in either type of measurement could also contribute. For example, differences of parameters such as the use of segmentation, the flip angle and the phase resolution might have affected the derived measurements of pulmonary regurgitation from the breath-hold and non-breath-hold acquisitions.

Regardless of the underlying mechanisms, the finding that pulmonary regurgitant fraction measurements may be lower when measured in held expiration than when measure during held inspiration, free breathing or by stroke volume difference remains important. For comparative studies, either serially or between patients, adherence to one type of acquisition and one type of breathing instruction are likely to be necessary.

Measurements of flow by phase velocity mapping can be subject to errors, for example due to eddy currents and uncorrected Maxwell gradients, especially when gradients are switched rapidly to achieve short echo times for breath-hold acquisition [16,17]. Such errors are likely to affect the breath-hold more than the non-breath hold acquisitions. The relatively poor temporal resolution in acquisition of the former relative to the latter may also be relevant. However, the Siemens Sonata system that we used incorporated software to correct for Maxwell gradients, and both of the breath-hold acquisitions used identical sequences and image orientation, and almost identical location, moved only minimally according the respective inspiratory or expiratory position of the pulmonary trunk. It is therefore unlikely that methodological differences could account for the differences between the inspiratory and expiratory breath-hold measurements.

In our centre we have used expiratory breath-hold velocity acquisitions for measurements of pulmonary regurgitation for over five years. Initially this was as an adjunct to non-breath-hold acquisitions, but subsequently as our preferred approach, given the advantages of time-saving and avoidance of respiratory motion related artefacts. However, we do not feel that a recommendation can be made for all centres or CMR systems, except that methodological consistency is important. Users should be aware of the potential affect on measurements should a transition be made from one method to another. The method used for a particular measurement of pulmonary regurgitation should be stated when the result is reported.

## Competing interests

The authors declare that they have no competing interests.

## Authors' contributions

BJ participated in the study, analysed the data and drafted the manuscript. SVB-N acquired the data and, with PJK, conceived, designed and coordinated the study. All authors contributed to, read and approved the final manuscript.

## References

[B1] Murphy JG, Gersh BJ, Mair DD, Fuster V, McGoon MD, Ilstrup DM, McGoon DC, Kirklin JW, Danielson GK (1993). Long-term outcome in patients undergoing surgical repair of tetralogy of Fallot. N Engl J Med.

[B2] Norgaard MA, Lauridsen P, Helvind M, Pettersson G (1999). Twenty-to-thirty-seven-year follow-up after repair for Tetralogy of Fallot. Eur J Cardiothorac Surg.

[B3] Gatzoulis MA, Balaji S, Webber SA, Siu SC, Hokanson JS, Poile C, Rosenthal M, Nakazawa M, Moller JH, Gillette PC, Webb GD, Redington AN (2000). Risk factors for arrhythmia and sudden cardiac death late after repair of tetralogy of Fallot: a multicentre study. Lancet.

[B4] Redington AN (2006). Determinants and assessment of pulmonary regurgitation in tetralogy of Fallot: practice and pitfalls. Cardiol Clin.

[B5] Chaturvedi RR, Redington AN (2007). Pulmonary regurgitation in congenital heart disease. Heart.

[B6] Therrien J, Marx GR, Gatzoulis MA (2002). Late problems in tetralogy of Fallot – recognition, management, and prevention. Cardiol Clin.

[B7] Bouzas B, Kilner PJ, Gatzoulis MA (2005). Pulmonary regurgitation: not a benign lesion. Eur Heart J.

[B8] Rebergen SA, Chin JG, Ottenkamp J, Wall EE van der, de Roos A (1993). Pulmonary regurgitation in the late postoperative follow-up of tetralogy of Fallot. Volumetric quantitation by nuclear magnetic resonance velocity mapping. Circulation.

[B9] Babu-Narayan SV, Kilner PJ, Li W, Moon JC, Goktekin O, Davlouros PA, Khan M, Ho SY, Pennell DJ, Gatzoulis MA (2006). Ventricular fibrosis suggested by cardiovascular magnetic resonance in adults with repaired tetralogy of fallot and its relationship to adverse markers of clinical outcome. Circulation.

[B10] Biancia CD, Yorke E, Chui CS, Giraud P, Rosenzweig K, Amols H, Ling C, Mageras GS (2005). Comparison of end normal inspiration and expiration for gated intensity modulated radiation therapy (IMRT) of lung cancer. Radiother Oncol.

[B11] Chen JH, Chai JW, Chu WC, Chang JM, Shen WC, Lee SK (2002). Free breathing magnetic resonance cholangiopancreatography (MRCP) at end expiration: a new technique to expand clinical application. Hepatogastroenterology.

[B12] Wallerson DC, Devereux RB (1987). Reproducibility of echocardiographic left ventricular measurements. Hypertension.

[B13] Presson RG, Audi SH, Hanger CC, Zenk GM, Sidner RA, Linehan JH, Wagner WW, Dawson CA (1998). Anatomic distribution of pulmonary vascular compliance. J Appl Physiol.

[B14] Ley S, Fink C, Puderbach M, Zaporozhan J, Plathow C, Eichinger M, Hosch W, Kreitner KF, Kauczor HU (2006). MRI Measurement of the hemodynamics of the pulmonary and systemic arterial circulation: influence of breathing maneuvers. AJR Am J Roentgenol.

[B15] Gatzoulis MA, Clark AL, Cullen S, Newman CG, Redington AN (1995). Right ventricular diastolic function 15 to 35 years after repair of tetralogy of Fallot. Restrictive physiology predicts superior exercise performance. Circulation.

[B16] Gatehouse PD, Keegan J, Crowe LA, Masood S, Mohiaddin RH, Kreitner KF, Firmin DN (2005). Applications of phase-contrast flow and velocity imaging in cardiovascular MRI. Eur Radiol.

[B17] Kilner PJ, Gatehouse PD, Firmin DN (2007). Flow measurement by magnetic resonance: a unique asset worth optimising. J Cardiovasc Magn Reson.

